# Lord Lister: V. His Early Researches and Conclusions

**Published:** 1918-02-23

**Authors:** 


### LORD LISTER.

### V.—His Early Researches and Conclusions.

In the previous paper we gave a sketch of the practice in operating that prevailed at the time, and for a good many years after the time, when Lister was appointed to the professorship of clinical surgery at Glasgow, and of the terrible mortality that resulted from what were known as "hospital diseases," which included pyæmia, septicæmia, erysipelas, especially an erysipelatous inflammation that invaded the fasciæ and inter-muscular tissues, and went by the name of cellulitis; hospital gangrene, and tetanus. The first three were endemic, the last two appeared occasionally only, but when they did appear, assumed an epidemic spread, and caused a frightful rate of mortality. The result of an operation was the merest lottery, and could never be predicted. It seemed to be a mere chance whether the patient recovered or whether he was attacked by one of these diseases, which no care, no skill, no assiduity, no precautions, in the state of knowledge then existing, was enough to avert. A mortality rate of 25 per cent. of the amputations of all kinds was considered a rate to be proud of, and was much exceeded in many hospitals; and the worst and most disheartening feature in surgery was, as has been said, the utter uncertainty of the result of an operation. Any operation, even one which, as far as the size of the incision was concerned, appeared to be trivial, might be fatal, and the fatal consequence occurred capriciously, and had no known relation to any factor in the operation. This being the prevailing practice, and this the prevailing expectation as to result, let us now inquire what the prevailing state of knowledge was.

### Lister's Starting Point.

There was, in fact, very little knowledge, and as is always the case where there is very little knowledge, there was much conjecture and much dogmatism. The less men know, the more cocksure they are. A few data stood out prominently as grounds for conjecture. The first was that hospital diseases were, as their name implies, endemic in hospitals, and in some way connected with the association of many patients together within the hospital walls. The second was that the diseases appeared to be infectious. They spread from patient to patient. If one patient in a ward was attacked, other patients in the same ward were sure to be attacked; and the certainty appeared to be the greater the more closely the patients were packed together. As we have already seen, the infection was supposed to cling to the walls and fabric of the hospital, which appeared to be saturated with it; and, above all, it was universally assumed that the infection was conveyed through the air, that it was in the air, that contact of the air with the wound produced the diseases. This preconceived conviction that the infection is in the buildings and is conveyed by the air permeated and saturated all the conjectures and reasonings on the subject, and was the chief cause of the long delay in effecting any improvement. It was with this conviction in his mind that Lister started on his investigations, and simple as the matter seems to us now that he has got to the bottom of the mystery and explained it all to us, we shall never understand the course of his investigations and the slow and gradual attainment of his conclusions unless we keep constantly in mind those fixed data from which he started, that the diseases are infectious, and that the infection is conveyed through the air; and not only that it was conveyed through the air, but that it was conveyed through the air alone, and in no other way. It was on this latter supposition that all the early experiments of Lister were conducted, and it was not until this supposition was discarded that progress was made by leaps and bounds.

### "Something in the Air."

The air is constituted, as we have known since the days of Priestley, of oxygen and nitrogen, and even as early as the eighteenth century John Hunter, and very early in the nineteenth century John Abernethy, had come to the conclusion that the cause of these and other inflammations was not mere contact with the gases of the air, for in surgical emphysema, where air is diffused widely through the cellular tissue, and may diffuse itself all over the body, and even into the limbs, inflammation does not follow. These early and clear-sighted investigators saw clearly that the cause of inflammation could not be contact with the normal constituent gases of the air, but the clearness of their vision was not possessed by their contemporaries or by their successors. The general opinion was not that the infection was in the gases of the air, or that it was not, but that somehow, in some vague way, the air had something to do with it; and more that it was "something in the air" than that it was the air itself. The writer remembers that in 1865 that accomplished physician, Dr. Julius Pollock, expressed to him in conversation that the infection consisted in "minute fungoid growths," a marvellously good shot, and probably the result of a knowledge of Pasteur's researches; but Pollock was before his time.

The firm belief that the infection is in the air and is conveyed by the air led, as beliefs always do lead, to practice; but by no means to consistent practice. One result of the belief was the practice of excluding access of the air to recently inflicted wounds wherever it was practicable to do so. For instance, the small wound made by the broken end of a tibia penetrating the skin was sealed, after the fracture had been reduced, with collodion or

☛ Previous articles appeared January 26, p. 351, February 2, p. 371, 9, p. 393, and 16, p. 415.

**LORD LISTER**—(*continued*).

merely with a pad of lint soaked in the blood that oozed from the wound; and the practice was sometimes successful. It was successful when the protruding end of the bone had escaped pollution and when the surgeon had refrained from the common practice of groping into the wound with his finger in order to discover the exact extent of the injury. The sealing of operation wounds was seldom attempted unless they were small, for in the first place air must have gained access to the wound during the operation, and before the sealing could take place the mischief was already done; and in the second place, very few surgeons appreciated the importance of a dry wound. Spouting arteries were tied, but no attempt was made to stop hæmorrhage altogether before closing the wound, and consequently after it was closed blood accumulated in the interior, and as the surface of the wound had always been contaminated to some extent suppuration always followed, pus accumulated in the interior, and the blood and pus must be let out. It required very little experience to discover that if they were not let out, not only were the surfaces of the wound kept apart and healing rendered impossible, but also absorption of what were called "the products of decomposition" took place and fever ensued, which was always mitigated by letting out the accumulated discharges. To prevent this absorption and the consequent fever it became the practice to facilitate the escape of the discharges—the blood and pus—by inserting drainage tubes; and to prevent the accumulation by hastening the adhesion of the surfaces of the wound to one another it became the practice to press these surfaces together by means of pads held in place by strapping and bandages. The lips of the wound were stitched together and its external surface covered by some dressing or other—plain water dressing that is to say, but dipped in tap water or Friars' balsam, lead lotion, or some other appliance, such as a poultice or what not. Every surgeon had his own favourite dressing. This was the orthodox practice, but some surgeons resorted to what was called "the open method"—that is to say, they made no attempt to close the wound, but left its surfaces gaping, in order to allow the freest possible escape for discharges; nor did they make any attempt to exclude the fatal air from the wound; and yet some of their cases escaped the fatal hospital diseases, though, of course, as the wounds must heal, if at all, by granulating from the bottom, the process was extremely slow.

This was the state of knowledge, these were the practices in vogue, and these the results attained when Lister began his researches. Suppuration was the natural and practically inevitable result of wounds; besides suppuration, hospital diseases were extremely frequent and extremely fatal. They were produced by the infection of a miasma carried by the air, and there was no known method and scarcely an attempt made either to purify the air or to prevent its access to the wound. The possibility that the infection might be carried from patient to patient by surgeon or dresser or nurse or instrument never seems to have occurred to anyone, and it was long before it occurred to Lister. The air was the sole culprit.

Lister's very earliest researches, in 1855, immediately after he went to Edinburgh, were into the nature of inflammation, for he recognised what few medical or surgical students then recognised, that the process of inflammation lay at the root of surgery, and was closely akin to the process of healing of wounds. When a wound was made, it took of necessity one of two courses. Either it healed at once when the raw surfaces were put together, or, in the great majority of cases, it suppurated; and suppuration is the result of inflammation. If, then, we could prevent inflammation, we could prevent suppuration and secure the healing of every wound by first intention. This was Lister's reasoning, and it is impeccable. But if we would prevent inflammation, we must first study it to determine its nature and causes. Hence Lister's earliest research was into the nature of inflammation.

### Ten Years' Work.

Ten years afterwards he had felt his way, slowly and cautiously, but surely to the following conclusions:

First: That hospital diseases occurred from infection of wounds.

Second: Infection is due to contact of the air with the wound.

Third: The infection is not the air itself, but something in the air.

Fourth: Infection does not occur unless the wound is suppurating.

Fifth: Suppuration is due to the decomposition or putrefaction of the discharges in the wound.

Sixth: That this decomposition or putrefaction also is due to infection by something in the air.

The practical conclusion from these data is manifest enough. It is that the substance or substances in the air, whatever they may be, that cause decomposition of the discharges in the wound, must be prevented from gaining access to the wound. If only this could be done, not only would infection of hospital diseases be prevented, but suppuration also would be prevented, with all its dangers of infection, all its debilitation of the patient, all its delay in the healing of the wound; and the severest and most extensive amputation wound could be compelled to heal by first intention and in a few days, even in a hospital, as surely as, nay, far more surely than, a trifling cut on a finger would heal by first intention in the pure air of the country or far out at sea.

The prospect might well seem chimerical, and did seem to the surgeons of those days utterly chimerical; but Lister clung to his dream. He set it before him as an ideal, and in the fulness of time he attained it. Through good report and ill report, through failures and disappointments and weariness; through ridicule and scorn; through jealousy and misrepresentation, he clung to his ideal; and in due time he attained it, and in attaining it conferred incalculable benefit upon the human race.

